# The Current Status and Future Promise of SPR Biosensors

**DOI:** 10.3390/bios12110933

**Published:** 2022-10-27

**Authors:** Nan-Fu Chiu

**Affiliations:** 1 Laboratory of Nano-Photonics and Biosensors, Institute and Undergraduate of Electro-Optical Engineering, National Taiwan Normal University, Taipei 11677, Taiwan; nfchiu@ntnu.edu.tw; Tel.: +886-2-77496722; 2 Department of Life Science, National Taiwan Normal University, Taipei 11677, Taiwan

The most commonly used protein detection methods in clinical diagnosis and disease monitoring are enzyme-linked immunosorbent assay (ELISA), Western blotting (immunoblot), and lateral flow assay (LFA) rapid screening, of which ELISA is the gold standard immunoassay in clinical practice. Biosensors can be used for the early diagnosis and monitoring of infectious diseases, cancer, and pathological conditions, and they can significantly improve the prognosis and survival. Biosensors represent an excellent analytical tool to study potential biomarker candidates and analyze the affinity of two molecules for each other. However, current assay technology is rapidly changing with the development of novel and diverse biosensors. The development of these novel biosensor technologies is influenced by the breakthrough and controllability of molecular medicine, nanotechnology, and nanofabrication, which can improve the quality of medical care and increase diagnostic accuracy. These novel sensing technology principles can be classified into fields including electrochemistry, mechanics (mechanical energy), electric, piezoelectric, acoustics, and optics etc. [1]. Over the past 30 years, the application of these bioassay techniques has grown rapidly in biosensor devices to monitor rapid, highly specific biomolecular recognition events. As of 2019, more than 55,971 studies related to biosensors have been published [2]. Among them, optical sensing is regarded to be the most advanced diagnostic technology, including fluorescence, surface plasmon resonance (SPR), Raman mass spectrometry, and terahertz wave technologies.

In this Special Issue, we focus on SPR biosensors, discuss the principles of this SPR technology and its future development, application, and importance. SPR has been shown to be one of the most versatile frameworks for the application of biosensors in different scientific fields. SPR biosensors have a variety of applications in life sciences, therapeutic drug monitoring, quality control, food, gases, chemical vapor, environmental testing, and more. SPR is a biosensing technology for the real-time assay of biomolecular binding interactions, and SPR biosensors are used for the label-free detection of various classes of biomarkers, with the advantages of simple operation, fast response, and high selectivity. SPR technology can quickly track molecular interactions in real-time diagnostics, and it is a powerful and widely used biological and chemical sensing technology. SPR is used to monitor binding events between biomolecules, ranging from cells, exosomes, proteins, aptamers, peptides, and nucleic acids to small molecules used in chemistry and pharmaceutics. Another common technique, biolayer interferometry (BLI), has the same optical abilities as SPR and is used in biophysical techniques to detect molecular binding interactions; however, SPR is the most common, stable, and widely used technology with a large sensing area and multi-channel application.

Compared with the traditional immunoassay ELISA, SPR can provide fast, real-time affinity, and/or kinetic data. Consequently, the advantages of SPR detection technology over ELISA have been proposed to include real-time monitoring, label-free detection, small sample size, reusable sensor chips, the easy analysis of complex samples, molecular interactions, reduced consumable costs, easier operation, and shorter experimental time.

SPR is an important sensing technology in biosensors that can be modulated by materials and optical systems to improve sensing sensitivity. In particular, the sensitivity of biosensors can be enhanced by the interfacial electric field modulation of SPR, such as in prism-based SPR biosensors [3,4], fiber-based SPR biosensors [5,6], grating-based SPR biosensors [7,8], 2D materials-based SPR biosensors [9,10,11], plasmon-enhanced fluorescence [12,13,14], and surface-enhanced Raman spectroscopy [15,16,17]. The enhancement of fluorescence and Raman signals is based on an increase in the local electric field on the nanostructure surface. The increase in the local electric field is caused by local plasmon resonance and evanescent field effects. When the excitation light approaches the resonance frequency of the nanostructure, the localized surface plasmon effect generates a strong local evanescent electric field on the surface and edges of the structure. Therefore, enhancing the interface electric field effect improves the sensing sensitivity.

This Special Issue brings together outstanding research on SPR biosensors, highlighting SPR as an emerging research topic ranging from biomedical fields (early diagnosis to treatment monitoring) to food quality control and environmental analysis. SPR biosensors for biomedical applications are receiving increasing attention in the scientific community. From the perspective of precision medicine, point-of-care analytics, and personalized pharmacology, it is a challenge to replace existing bulky and expensive instruments with smart sensors of smaller size, lower cost analytical systems, lab-on-a-chip systems, and paper-based devices. The rapid development of SPR biosensors, structural design concepts, fabrication techniques, sensing materials, numerical simulations, gas sensing, and diagnostic applications will help to significantly improve the accuracy and sensitivity of detection in future clinical applications [18,19,20].

In addition, SPR is an excellent analytical technique to monitor the kinetics of molecular interactions and a key factor for determining kinetic constants for this method of interaction mechanics is that the concentration of free protein in the molecule to be tested should rapidly equilibrate with the flowing solution. If the association reaction between molecules is much faster than mass transfer, the binding reaction is considered to be limited by mass transfer. Therefore, the interaction kinetics are considered to be correct if mass transport is fast and the association between molecules is slow. Consequently, the molecular mass transfer rate is a key factor that must be considered. To minimize mass transport limitations, SPR assays using higher flow rates (≥30 μL/min) and lower surface densities of immobilized ligands are more beneficial for the assessment of molecular interactions. However, the modulation of refractive index or thickness based on metal surfaces and novel materials can enhance the sensitivity of SPR sensors for the direct detection of small molecules (<10 kDa) or ultra-low concentrations (<1 pM) of analytes. These features allow for the further study of the interactions of challenging biomolecules.

In this Special Issue, Alavi et al. [21] report the SPR analysis of SUMO-Murine Rap1-Interacting Factor 1 C-Terminal Domain Interaction with G4. Previous studies have shown the role of mouse Rif1 (muRif1) C-terminal domain (CTD) in binding to G-quadruplexes (G4) and that SPR technology can be used to investigate Rif1 and G4 interactions. Therefore, Alavi et al. assessed its binding with G4 at nano-molar concentrations using SPR and found that muRif1-CTD had high affinity for this G4 sequence as it showed a very low KD (6 ± 1 nM). The authors studied muRif1-CTD (analyte) and G4 (ligand) interactions via SPR, which were shown to have high accuracy for ka and kd kinetic constants. These kinetic values are important not only to understand the mechanism of action of the interaction of this biomolecule with its partner but also for screening new drug candidates.

In this Special Issue, Wei et al. [22] investigated the rapid detection of virus nucleic acid via isothermal amplification on a plasmonic-enhanced digitizing biosensor. The development of rapid and accurate pathogen screening technology is of great significance for the early diagnosis and prevention of infectious diseases. In their study, the authors used a loop-mediated isothermal amplification (LAMP) SPR biosensor for the hepatitis virus. The detection principle was based on SPR-enhanced fluorescence. The target nucleic acid was amplified using modified LAMP with fluorescence resonance energy transfer (FRET-LAMP) primers. They successfully achieved the rapid detection of the hepatitis virus by integrating modified isothermal amplification to improve the signal contrast and assay time with a plasma-enhanced sensor. The results showed that hepatitis virus nucleic acid with a concentration of 10^−3^ to 10^−4^ mg/mL could be detected within 10 min. The authors concluded that they hope this technology can be further applied to the DNA screening of different pathogens in the future to achieve the rapid detection of diseases.

In this Special Issue, Sun et al. [23] utilized a SPR biosensor with magnetic sandwich hybrids for signal amplification. They proposed a method using target analytes which were captured by receptor-modified magnetic nanoparticles (MNPs), and then biotinylated recognition elements were attached to the analyte-bound MNPs to form a sandwich hybrid structure. This structure was directly delivered to a neutravidin-modified SPR fluidic channel. The MNP hybrids were then captured by the SPR chip through the neutravidin–biotin interaction, resulting in an enhanced signal. The performance of the magnetic-based SPR biosensor was evaluated by detecting DNA and Aβ40, in which the sandwich structure was directly delivered to the SPR chip surface down to 1 fM for DNA or 10 fM for Aβ40. This magnetic-based SPR biosensor method combined the benefits of a rapid response, real-time measurement, high sensitivity, and excellent specificity. This method will help to develop the new applications of SPR biosensors in the future, with the advantages of multiple signal amplification and magnetic preconcentration, which should be particularly valuable for the detection of small molecules and ultra-low concentration analytes.

In this Special Issue, Dr. Chang [24] performed a review of recent advancements in aptamer-based SPR biosensing strategies. Aptamer-based SPR biosensors have attracted significant attention because of their simplicity, feasibility, replacement of antibodies, and low cost for target detection. Dr. Chang hopes that this review will guide the development of SPR aptamer sensors for healthcare. The basic sensing mechanism of SPR aptasensors is similar to that of other SPR biosensors. The aptamers are immobilized on a gold film sensing surface, following which the aptamer probe recognizes and interacts with its target, resulting in detectable signals. Dr. Chang also reviewed the detection technology of aptamer-based SPR biosensors in different materials, such as MNPs, quantum dots (QDs), graphene, graphene oxide (GO), spherical gold nanoparticles (AuNPs), gold nanocages, and nanorods. In addition, Dr. Chang also reviewed the development of nucleic acid amplification detection technology in aptamer-based SPR biosensors in recent years. The review showed that detection technology using different acid detection amplification methods, such as rolling circle amplification (RCA), hybridization chain reaction (HCR), and catalytic hairpin assembly (CHA), combined with SPR detection technology could achieve a rapid diagnosis with highly sensitive and specific detection. The findings of this comprehensive review show that DNA aptamers and peptide aptamers have the characteristics of high specificity, easy storage, and high temperature resistance. Therefore, aptamer biosensors are expected to be excellent sensing probes and may also be used substitutes for antibodies in the field of biosensors.

This Special Issue includes pioneering work on SPR biosensor analysis, including small molecule interaction kinetics, LAMP-based SPR biosensors, MNP-based SPR biosensors, and aptamer-based SPR biosensors. The results demonstrate the potential of these SPR biosensors for label-free and real-time biosensing approaches in the detection of disease-related small-molecule metabolites, proteins, and nucleic acid amplification. The rapid development of the broad field of SPR biosensors will have a huge impact on the assay industry and medical treatment and increasingly impact and contribute to precision healthcare.
